# A national-scale sampled temperate fuel moisture database

**DOI:** 10.1038/s41597-024-03832-w

**Published:** 2024-09-06

**Authors:** Katy Ivison, Kerryn Little, Alice Orpin, C. H. M. Lewis, Niamh Dyer, Lily Keyzor, Luke Everett, Erin Stoll, Roxane Andersen, Laura J. Graham, Nicholas Kettridge

**Affiliations:** 1 School of Geography, Earth and Environmental Sciences, University of Birmingham, Birmingham, UK; 2 Environmental Research Institute, University of the Highlands and Islands, Thurso, KW147JD UK; 3 Biodiversity, Ecology and Conservation Group, International Institute for Applied Systems Analysis, Vienna, Austria

**Keywords:** Natural hazards, Hydrology

## Abstract

Fuel moisture content (FMC) is important for the ignitability, behaviour and severity of wildfires. Understanding the drivers of FMC and its spatial and temporal variability can help us develop fuel moisture models and inform assessments of wildfire behaviour and danger. Here we present the first United Kingdom (UK) national-scale temperate FMC dataset of 8,057 samples of eighteen different fuel constituents collected across 58 sampling sites between 2021–2023. We sampled fuels across emerging fire-prone ecosystems in the UK across three studies: (1) UK-wide longer-term sampling characterising the spatio-temporal drivers of FMC; (2) landscape-scale measurement through the North Yorkshire Moors to investigate landscape-driven variability in FMC; (3) plot-scale intensive sampling in the West Midlands to quantify diurnal patterns and among-sampler variability in fuel measurements. This database addresses a global fuel moisture measurement gap within traditionally non-fire prone regions. The database will advance our understanding of temperate fuel moisture dynamics and forms a fundamental contribution towards the development of a fire danger rating system for traditionally non-fire prone regions such as the UK.

## Background & Summary

Temperate regions which are not typically prone to wildfires are experiencing a greater number of wildfires due to climate and land use change^1,2^. The UK currently experiences around 30,000 wildfires per year, here defined as any uncontrolled vegetation fire which requires a decision or action regarding suppression^3^, resulting in a burned area of 6,600 hectares on average^3^. Fires can result in large economic costs (loss of crops, evacuation of houses, infrastructure closure) and ecological damage (e.g. loss of ground-nesting bird habitat)^4^, and fire-prone environments such as heathlands and peatlands often contain sequestered carbon stores in organic soils that can be released during severe wildfires^4^.

Wildfire behaviour is intrinsically linked to fuel moisture content (FMC). Specifically, fuel moisture largely influences ignitability, flammability, fire behaviour and associated severity of wildfire impacts^5,6^. Understanding the drivers of FMC is therefore critical to predict when wildfires might occur and to develop wildfire management strategies^4^.

FMC can be measured directly, or estimated using numerical models or remotely sensed data. For example, a number of models have been developed to predict FMC from weather observations such as temperature, humidity, precipitation and wind speed^7–10^. Such models were developed for dead fuels in traditionally fire prone regions such as in the pine forests of North America^7–10^ and Australia^11^ and have been applied globally with varying success^12,13^. Remote sensing can also be used to estimate FMC, particularly for live fuels (e.g. in the Mediterranean^14^, the United States^15,16^), although studies report some remaining challenges in the ability of remote sensing to accurately capture FMC^14–16^. The only direct measure of FMC is by destructive field sampling of fuel. This method is extremely labour-intensive and must be repeated over time and across a variety of locations to capture spatial and temporal variability^17^. Fuel moisture data are currently lacking in traditionally non-fire prone temperate regions, but are urgently needed to develop our understanding of spatio-temporal patterns in fuel moisture which will enable tailored moisture models to be created. Indeed, Globe-LFMC, the largest published database of sampled FMC, contains over 280,000 records of live FMC from eleven countries but only contains 24 samples from England and 250 samples from Scotland taken across six sites. In addition, the majority of these samples (~258,000), are taken from the predominantly western and southern US and Mediterranean France, leaving a large knowledge gap in temperate regions. The most commonly sampled species in this dataset are arid or semi-arid shrubs (*Adenostoma fasciculatum, Artemisia tridentata*) and pine species^17^. However, fire-prone ecosystems within the UK are mostly dominated by heather (*Calluna vulgaris*), gorse (*Ulex europeaus*), bracken (*Pteridium aquilinum*) and moor grass (*Molinia caerulea*). Within the UK, a number of studies have characterised different aspects of FMC; for example, to investigate the ignition thresholds and to characterise the spatial and temporal patterns of FMC for live heather^18,19^, and to look at the FMC of heather, litter and moss in relation to fire spread^20^. However, we lack information about FMC over a large spatial scale (e.g. national) and over a longer time period (e.g. of multiple years), and for many of the fuels found within the UK’s fire-prone environments (gorse, bracken, moor grass) there are currently no FMC data available.

Here, we present three datasets, associated with three separate studies, that aim to address these knowledge gaps. First we describe the UK-wide dataset, where sampling was conducted fortnightly to monthly across the UK over two years to examine wide-scale spatial and temporal patterns in FMC. Secondly we describe the North Yorkshire Moors (NYM) dataset, where intensive sampling was conducted over five days between April and July 2021 at 18 sites within the NYM area to examine cross-landscape variation in FMC. This dataset has been used to examine the landscape controls on fuel moisture^21^. Thirdly we describe the West Midlands (WM) dataset, where intensive sampling at two heathland locations was conducted during one day in a Country Park near Birmingham to characterise both plot-scale diurnal variability in FMC and among-sampler variability in fuel measurements^22^.

## Methods

### UK-wide dataset

**Fig. 1:**
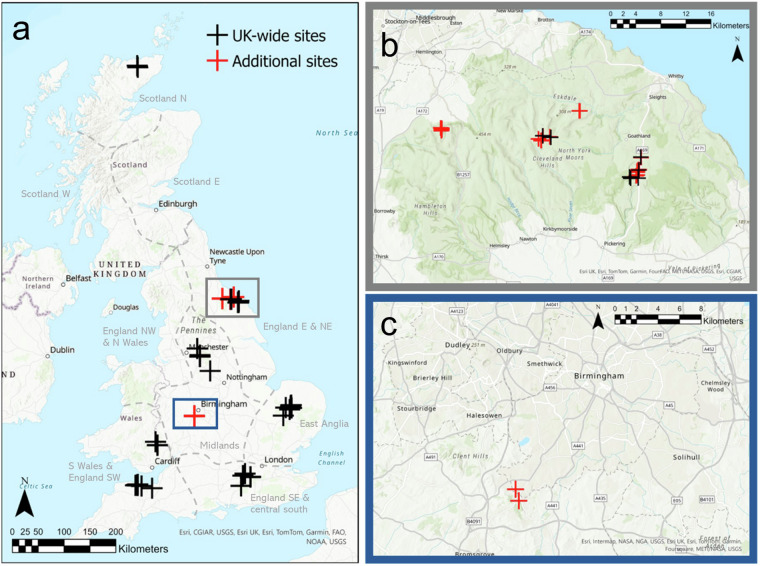
Location of sample sites (**a**). UK-wide sites were sampled regularly during 2021–2023. Additional sample sites underwent more concentrated sampling: in the North Yorkshire Moors (**b**), eighteen sites (five of which were also included in UK-wide dataset) were sampled extensively during spring and summer 2021; in Lickey Hills Country Park (**c**), two sites were sampled at different times on just one day. Sources: Esri, Airbus DS, USGS, NGA, NASA, CGIAR, N Robinson, NCEAS, NLS, OS, NMA, Geodatastyrelsen, Rijkswaterstaat, GSA, Geoland, FEMA, Intermap and the GIS user community. Grey dashed lines and associated grey labels show climate regions^24^, one of the key factors considered when choosing sampling locations.

We established fuel moisture monitoring sites at 43 heathland, bog, acid grassland and coniferous forest locations across the United Kingdom (marked in black in Fig. 1; Table 1). The monitoring sites were selected to encompass different land cover types according to the Land Cover Map^23^, climate regions of the UK^24^ and the range of soils^25,26^ within them. We endeavoured to represent the combinations of these factors of interest across our sites as much as possible to allow investigation of their influence on fuel moisture content.

**Table 1:** Overview of sites used in UK-wide, NYM and WM datasets.

Region	Site Location	No. sites	Land cover types
**UK-wide dataset sites**			
South West/South Wales	Holnicote Estate	6	Heathland, acid grassland, forest
	Croydon Hill	1	Forest
	Quantock Lodge	1	Forest
	Sugar Loaf Hill	2	Heathland, acid grassland
	Nant y Gwerydd	2	Heathland, forest
South East	Thursley Common	2	Heathland, forest
	Ockham Common	3	Heathland, forest
	Pirbright	2	Heathland, forest
	Cobham Common	1	Heathland
East Anglia	Milden Hall	1	Forest
	Lynford Hall	1	Forest
	Thetford	1	Forest
	Stanford Training Area	5	Heathland, acid grassland
Peak District	Matlock Moor	1	Forest
	Marsden Moor	1	Bog
	Hope Woodlands	4	Heathland, forest, acid grassland, bog
North Yorkshire Moors	Danby Estate*	2	Bog
	Goathland Moor*	1	Heathland
	Cropton	2	Forest
	Levisham*	2	Heathland
Scottish Highlands	Forsinain	1	Heathland
	Trantlemore	1	Heathland
**NYM dataset sites**	Levisham*	6	Heathland
	Danby Estate*	2	Heathland
	Lower Danby Estate	2	Heathland
	Rosedale Estate	2	Heathland
	Cringle Moor	5	Heathland
	Goathland Moor*	1	Heathland
**WM dataset sites**	Lickey Hills Country Park	2	Heathland

*denotes sites shared between datasets (in Levisham, an additional 4 sites were added to the NYM dataset in addition to the 2 sites used for the UK-wide dataset).

Eighteen different fuel constituents (Table 2) were collected at each site on a fortnightly basis from April to October in 2021 and 2022 and monthly from November to March. In 2023 samples were collected across seven days from February to June (Table S1; see supplementary file). These fuels are all found in abundance within fire-prone ecosystems within the UK. In all datasets (UK-wide, NYM and WM) we sampled fuel moisture content following a modified protocol from Norum and Miller^27^ that has been used for other fuel moisture campaigns in UK peatlands and heathlands^21,22^. For a full description of sampling protocol for the fuel types collected throughout all three studies see Little & Quiñones^28^. We established 20 m transects at sample sites and collected vegetation samples (heather, gorse, bracken, moor grass) between 11:00 and 17:00 local time. For heather, gorse and bracken we sampled stems and either canopy (heather, gorse) or leaves (bracken) separately, and for all vegetation both live and dead samples were taken. We also collected live moss samples (by pinching top two cm of moss from the soil surface) and litter samples (by pinching top two cm of dead leaf litter from soil surface) at ca. 10 points along this transect. These two fuel types were sampled separately, and if both occurred together the constituents were separated. For the organic layer, defined as the organic material beneath the surface litter and above the mineral soil, we sampled the top 5 cm of soil at four points along the transect. In forested sites, we collected twigs (<5 mm diameter) from fallen branches at ca. 10 points along the transect, only sampling those which were not in contact with the floor. We combined the same fuel constituents sampled along the transects within aluminium screw-fit tins that were sealed with masking tape. We calculated gravimetric fuel moisture content (Eq. 1) by weighing the collected samples, drying them for 48 h at 80 °C^27^ and then reweighing the dried samples. The FMC is calculated as:

1 $${\rm{FMC}}=({\rm{W}}-{\rm{D}})/({\rm{D}}-{\rm{T}})\ast 100$$

where W = wet weight (g), D = dry weight (g) and T = the weight of the sample tin (g). A total of 5,845 samples were collected.

**Table 2:** Fuel constituents sampled and the datasets within which they were collected.

Fuel constituents		Dataset
**Heather** (***Calluna vulgaris***)	Live canopy	UK-wide, NYM, WM
	Live stem	UK-wide, NYM, WM
	Dead canopy	UK-wide, NYM, WM
	Dead stem	UK-wide, NYM, WM
**Gorse** (***Ulex europaeus***)	Live canopy	UK-wide
	Live steam	UK-wide
	Dead canopy	UK-wide
	Dead stem	UK-wide
**Bracken** (***Pteridium aquilinum***)	Live leaves	UK-wide
	Live stem	UK-wide
	Dead leaves	UK-wide
	Dead stem	UK-wide
**Moor grass** (***Molinia caerulea***)	Live	UK-wide
	Dead	UK-wide
**Surface materials**	Litter	UK-wide, NYM, WM
	Moss	UK-wide, NYM, WM
	Organic layer	UK-wide, NYM, WM
	Twigs	UK-wide

Kestrel 3000 weather meters (Kestrel Instruments, Boothwyn, PA) were used to measure temperature, relative humidity and wind speed during sampling at each site. Daily mean temperature and daily precipitation were downloaded at 0.25 degree resolution (roughly 27 km at the equator) from the E-OBS ensemble gridded dataset version 26.0^29^ and these values were assigned to samples at the location and date of sampling. The number of days since precipitation occurred was calculated using these data. The R package ‘elevatr’^30^ was used to download elevation data, and the R package ‘raster’^31^ was used to download slope and aspect data for each site.

### NYM dataset

We chose eighteen heathland and peatland sample sites within the North Yorkshire Moors National Park in the northeast of England (Fig. 1b) that represented different soils (coarse, fine, peat^25^), aspect (north or south facing; these were chosen to account for the full range in solar radiation from maximum to minimum; OS Terrain® 50 DTM OS data © Crown copyright and database right 2022), and hillslope position (low (plateau below slope), medium (slope of hill) or high (top of plateau); OS Terrain® 50 DTM OS data © Crown copyright and database right 2022) (see Little *et al*.^21^). Each site contained a pair of plots, one with a mature heather canopy (last burned 15–20 years ago with an average height of 60 cm and accumulated moss/litter layer depth of 5 cm^32^) and a second with a building heather canopy (burned in the last 5–10 years with an average height of 30 cm and accumulated moss/litter layer depth of 2.5 cm^32^); canopy age was categorised based on land managers’ records^21^. Five of these sites were also used in the UK-wide dataset (Table 2), but samples were taken from these sites separately for each dataset. Sampling was carried out at all eighteen sites on each of the following dates between 11:00 and 17:00 local time: 15^th^, 18^th^ and 23^rd^ April, 13^th^ June and 22^nd^ July 2021. These dates were chosen as dry, hot days within the field season so that spatial patterns of FMC during high fire-risk periods could be discerned. In both this dataset and the WM dataset outlined below, seven fuel constituents were sampled: live heather canopy, live heather stems, dead heather canopy, dead heather stems, moss, litter and organic layer (Table 2). A total of 1,124 samples were collected.

### WM dataset

This study took place at two heathland sites within Lickey Hills Country Park, Birmingham (Fig. 1c) on 28^th^ March 2022. Sampling was carried out by seventeen different samplers every hour from 10:00 to 18:00 local time. A total of 1,088 samples were collected.

Finally, so that our datasets can be merged more easily with the existing Globe-LFMC dataset^17^, we provided the same meteorological and land cover data provided by Globe-LFMC. Meteorological data comprised AgERA5 (Agrometeorological indicators from 1979 to present derived from reanalysis)^33^ variables; 24 h mean and maximum air temperature, 24 h mean dewpoint temperature, 24 h summed precipitation, 24 h mean relative humidity, 24 h mean vapour pressure and 24 h mean wind speed. Land cover data was assigned using the IGBP classification from LP DAAC MCD12Q1.061 (MODIS/Terra + Aqua Land Cover Type Yearly L3 Global 500 m SIN Grid)^34^.

## Data Records

**Fig. 2:**
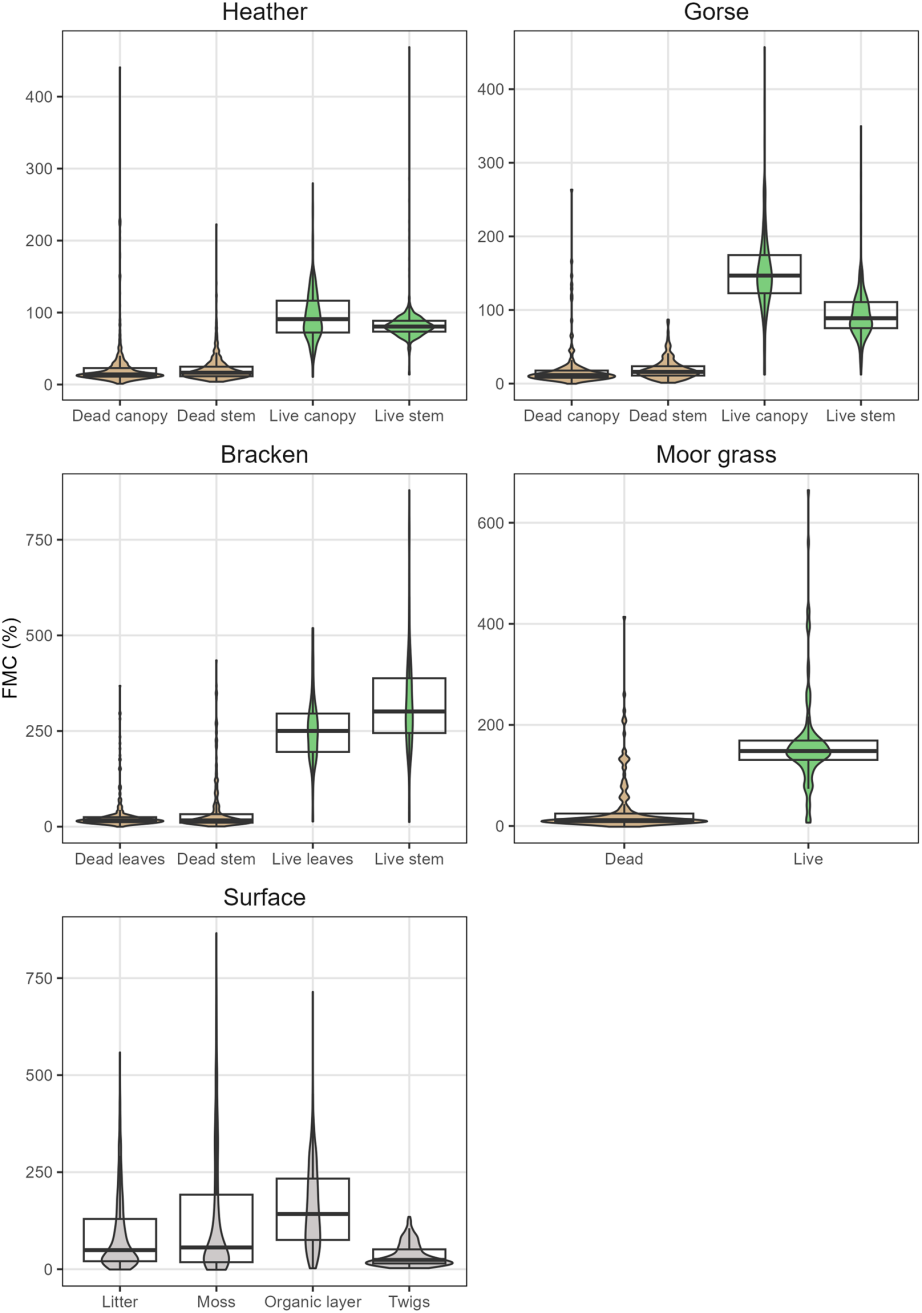
Range in fuel moisture content (FMC, %) of eighteen different fuel types sampled from UK-wide, NYM and WM datasets from 2021–2023. Brown violin plots = dead fuels, green = live fuels and grey = surface fuels. Median values and interquartile ranges are shown with overlaid boxplots. Note different y-axes.

**Fig. 3:**
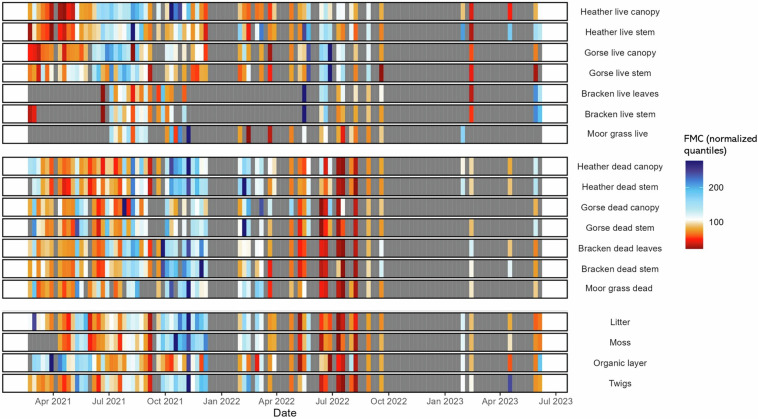
Fuel moisture content (FMC) of eighteen different fuel constituents averaged weekly during sampling period of 2021–2023. Weekly averages were calculated across all sample sites (UK-wide dataset, NYM dataset and WM dataset) and each week is represented by a coloured band. FMC across all fuel constituents was re-scaled using quantile normalization, a technique to standardise the statistical properties (e.g. quantiles) of multiple datasets to allow comparison between data originally on different scales. We can therefore see in which time periods FMC was lower (red) or higher (blue) across all fuels. Although FMC is measured as a percent, quantile normalization means that the legend scale is not representative of the true FMC values. Fuels have been grouped into live, dead and surface fuels to allow easier comparison between fuel types.

**Fig. 4:**
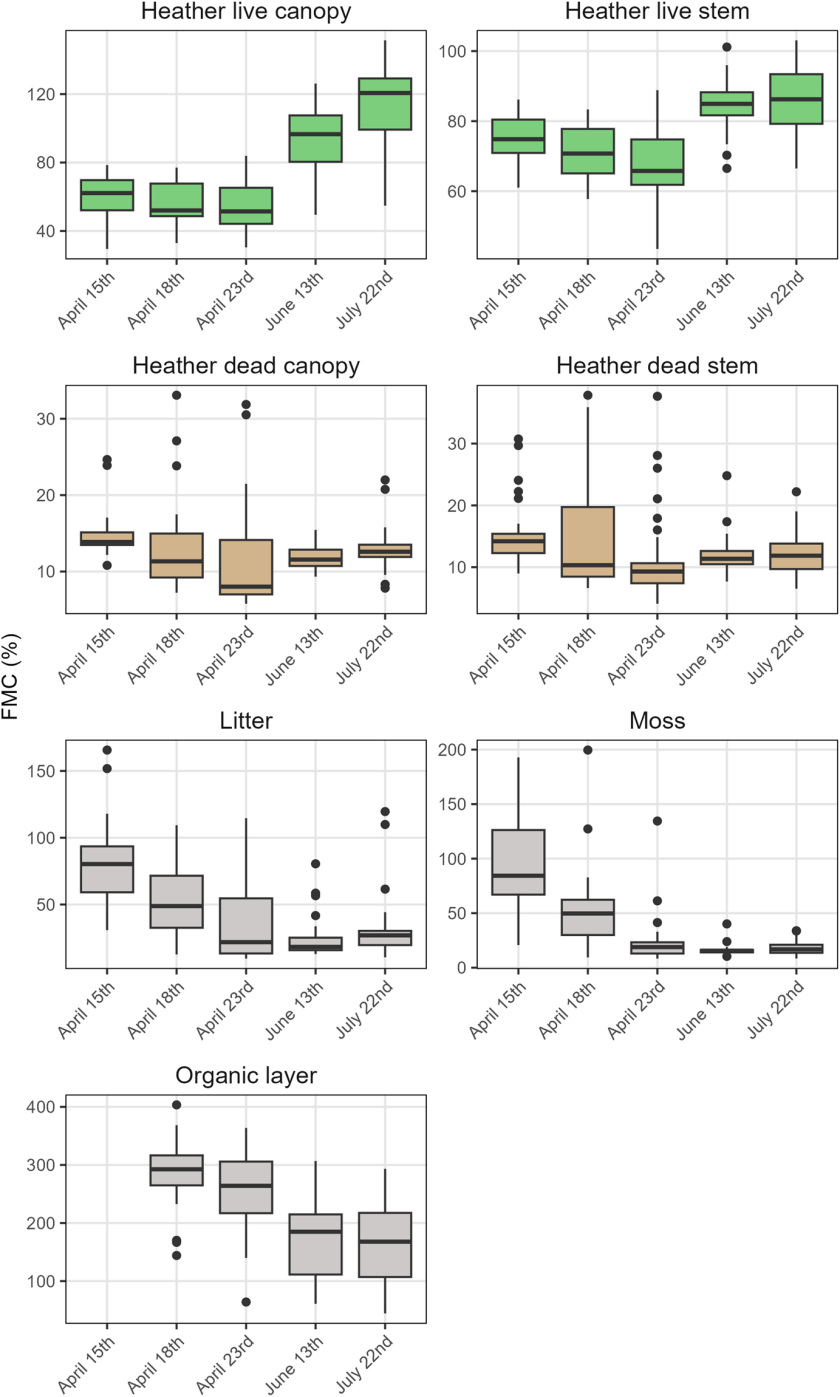
Variation in fuel moisture content (FMC) of seven fuel constituents in the North Yorkshire Moors, intensively sampled over 3 days in April and one day each in June and July. No samples were taken for the organic layer on April 15th. Note different y-axes.

**Fig. 5:**
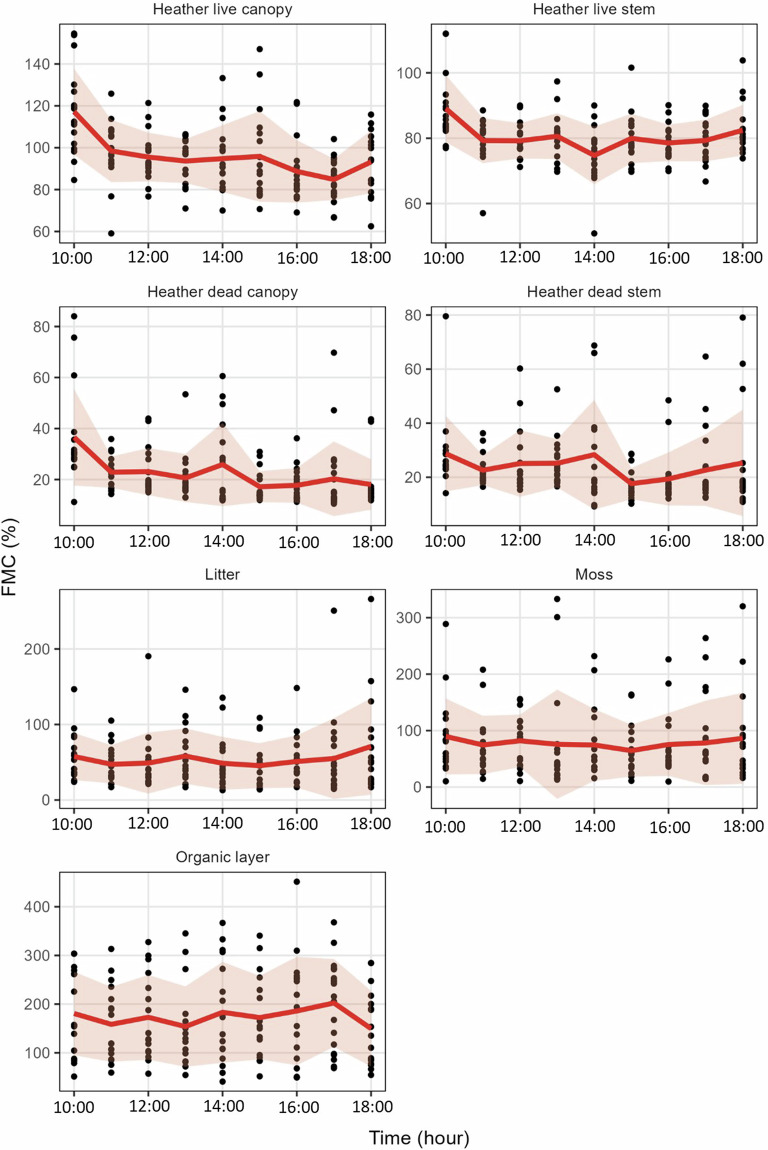
Fuel moisture content (FMC) of seven fuel constituents in the WM dataset, sampled from Lickey Hills Country Park, Birmingham, on 28th March 2022 over eight hours. Red lines represent mean FMC at each time, pink ribbons represent standard deviation of means. Note different y-axes due to large variation in FMC ranges between fuels.

The dataset repository^35^ contains three .csv files, one for each dataset; please see descriptions below for information about each. Due to differences in the content, scope and purpose of the three datasets we have presented them separately. Figures 2–5 and Tables 3–5 summarise the data collected.

**Table 3:** Summary of fuel moisture content (FMC, %) for each fuel type sampled in the UK-wide dataset.

Fuel type	No. samples	Mean FMC	Std. dev. FMC	Minimum FMC	Maximum FMC	FMC range	CV
Bracken live stem	157	325.86	139.54	12.1	879.66	867.56	42.82
Bracken live leaves	156	253.48	87.33	13.43	519.21	505.77	34.45
Moss	541	173.47	185.83	−1.31	865.97	867.28	107.12
Moor grass live	123	163.13	94.24	6.66	664.53	657.88	57.77
Gorse live canopy	201	151.31	55.24	12.2	457.14	444.94	36.5
Organic layer	667	144.83	108.06	2.38	714.69	712.31	74.62
Litter	806	105.85	107.11	−0.71	558.62	559.33	101.19
Heather live canopy	389	105.62	38.43	10.64	279.69	269.05	36.39
Gorse live stem	200	96.13	38.82	12.24	349.78	337.54	40.39
Heather live stem	389	86.03	30.53	13.88	468.8	454.92	35.49
Bracken dead stem	316	43.18	69.62	0.7	434.89	434.19	161.22
Twigs	265	35.89	28.9	2.83	134.92	132.09	80.54
Moor grass dead	193	32.74	54.76	−1.43	413.82	415.25	167.27
Bracken dead leaves	323	28.59	44.86	−0.1	368.26	368.36	156.88
Heather dead canopy	374	27	43.4	1.1	440.7	439.59	160.73
Heather dead stem	380	24.5	21.84	3.91	222.6	218.69	89.17
Gorse dead canopy	186	19.46	30.4	−0.19	263.53	263.72	156.21
Gorse dead stem	183	19.45	14.19	1.16	86.98	85.83	72.95

Some minimum FMC are less than 0; a threshold below 0 (−2% FMC) was selected as a cutoff to account for potential error in scales used (see Technical Validation). CV is calculated as (standard deviation/mean) * 100. This allows comparison of variation between fuel layers despite the large variety in FMC of different fuels. Fuel types have been ordered from highest to lowest mean FMC.

**Table 4:** Summary of no. samples and FMC (%) in NYM dataset for each sample day.

Fuel	Date	No. samples	Min. FMC	Max. FMC	CV
Heather live canopy	April 15th	36	29.52	78.50	19.11
	April 18^th^	33	32.96	77.02	22.27
	April 23^rd^	36	30.44	83.91	23.87
	June 13^th^	36	49.48	126.14	21.73
	July 22nd	36	54.80	151.37	22.06
Heather live stem	April 15th	36	61.01	86.15	8.64
	April 18^th^	34	57.76	83.34	10.32
	April 23^rd^	36	43.51	88.84	16.56
	June 13^th^	36	66.50	101.15	8.28
	July 22nd	36	66.49	103.06	10.78
Heather dead canopy	April 15th	20	10.81	24.66	23.24
	April 18^th^	27	7.23	33.08	46.18
	April 23^rd^	33	5.79	31.87	59.37
	June 13^th^	34	9.34	15.45	12.68
	July 22nd	28	7.83	21.99	23.36
Heather dead stem	April 15th	29	8.98	30.74	35.00
	April 18^th^	32	6.61	37.83	57.62
	April 23^rd^	35	4.08	37.66	64.23
	June 13^th^	36	7.67	24.81	23.82
	July 22nd	35	6.51	22.20	27.83
Litter	April 15th	18	30.79	165.64	42.99
	April 18^th^	33	12.62	109.38	52.36
	April 23^rd^	35	9.50	114.67	82.11
	June 13^th^	35	12.90	80.45	61.41
	July 22nd	34	10.47	119.59	74.79
Moss	April 15th	33	20.76	192.85	46.25
	April 18^th^	32	9.44	199.59	68.26
	April 23^rd^	35	8.35	134.51	95.08
	June 13^th^	35	10.41	40.14	29.54
	July 22nd	32	8.44	33.89	36.16
Organic layer	April 15th	0	NA	NA	NA
	April 18^th^	34	144.09	403.44	19.42
	April 23^rd^	32	63.99	363.77	27.76
	June 13^th^	36	60.90	306.75	37.46
	July 22nd	36	44.37	293.31	38.75

CV is calculated as (standard deviation/mean) * 100. This allows comparison of variation between fuel layers despite the large variety in FMC of different fuels.

**Table 5:** Mean FMC (%) for each fuel layer in WM dataset.

Fuel layer	No. samples	Overall mean FMC	10:00	11:00	12:00	13:00	14:00	15:00	16:00	17:00	18:00	FMC range	CV
Heather live canopy	155	22.46	117.20	98.45	95.52	93.62	94.77	95.83	88.67	84.82	93.19	23.57	9.40
Heather live stem	155	23.88	89.07	79.27	79.21	80.60	74.74	79.94	78.56	79.31	82.44	14.33	4.80
Heather dead canopy	156	95.58	36.58	22.88	23.11	20.63	25.89	17.19	17.71	20.28	18.05	19.38	26.76
Heather dead stem	156	80.33	28.71	22.59	25.07	25.15	28.34	17.62	19.35	22.63	25.27	11.09	15.62
Litter	155	53.72	57.65	47.34	48.85	58.18	48.63	45.50	51.06	54.95	70.98	12.68	14.72
Moss	156	77.81	89.57	74.51	81.87	75.69	74.44	64.38	75.31	78.17	86.25	25.19	9.53
Organic layer	155	173.83	180.67	158.46	172.91	153.90	183.19	172.30	185.89	202.39	149.69	29.29	9.81

Range is the difference between the highest and lowest mean FMC at any time. CV is calculated as (standard deviation/mean) * 100. This allows comparison of variation between fuel layers despite the large variety in FMC of different fuels. Columns labelled with hour of the day (e.g. 10:00) show mean FMC at that time.

The UK-wide dataset contains the following information: Date, Site name, Longitude (WGS84 decimal degrees), Latitude (WGS84 decimal degrees), Region of the UK (defined as separate regions using the Met Office Climate Districts Map^24^ but with some names changed, e.g. ‘Midlands’ (Fig. 1a) is named ‘Peak District’ in dataset, ‘England E & NE’ (Fig. 1a) is named ‘North York Moors’ in dataset), Land Cover Map^23^ land cover type, Elevation (m), Slope (degrees), Aspect (degrees), Soil type^25^, Air temperature at the time of sampling (degrees Celsius), Relative humidity at the time of sampling (%), Wind speed at the time of sampling (m/s), Mean 24 h air temperature (downloaded from E-OBS; degrees Celsius), 24 h precipitation sum (downloaded from E-OBS; mm/day), Number of days since rain, Fuel type (e.g. heather live canopy), Species name (taxonomic Latin names for heather, gorse, bracken and moor grass; NA for moss, litter, twigs and the organic layer; moss and twig species were not identified), Time collected (in hours), FMC (fuel moisture content in %) and Outliers removed (Y if discarded from analyses as an outlier, N if retained for analyses; meteorological data (E-OBS and AgERA5) were not assigned to outliers).

The NYM dataset contains the following information: Date, Site name, Longitude (WGS84 decimal degrees), Latitude (WGS84 decimal degrees), Fuel type (e.g. heather live canopy), Species name (taxonomic Latin name for heather samples, NA for moss, litter and the organic layer; moss species were not identified), landscape characteristics of the sample sites comprising Heather canopy age (mature or building (i.e. growing)), Soil texture (coarse, fine or peat), Hillslope position (low, medium or high) and Aspect (north or south) and FMC (fuel moisture content in %).

The WM dataset contains the following information: Date, Sampler (containing IDs of 17 samplers from A to Q), Site name (either north or south sites within the park), Longitude (WGS84 decimal degrees), Latitude (WGS84 decimal degrees), Fuel type (e.g. heather live canopy), Species name (listed in the same way as for NYM dataset), Time collected (in hours from 1000 to 1800) and FMC (fuel moisture content in %).

All three datasets also contain the following weather variables from AgERA5^33^: 2 m air temperature (24 h mean, Kelvin (K)), 2 m air temperature (24 h maximum, K), 2 m dewpoint temperature (24 h mean, K), 10 m wind speed (24 h mean, m/s), vapour pressure (24 h mean, hPa), 2 m relative humidity at 6 h (%), 2 m relative humidity at 9 h (%), 2 m relative humidity at 12 h (%), 2 m relative humidity at 15 h (%), precipitation 24 h sum (mm/day), precipitation sum for 3 days before (mm/day), precipitation sum for 1 week before (mm/day), precipitation sum for 4 weeks before (mm/day) and precipitation sum for 12 weeks before (mm/day). Finally, the three datasets contain IGBP land cover ID and IGBP land cover type^34^.

## Technical Validation

All sample tins were given a unique identifier, which were recorded carefully to ensure that their weights were assigned to the correct location and date. To ensure that FMC was as accurate as possible, each empty sample tin was weighed individually before sampling to include in the FMC calculation (i.e. we did not use a mean tin weight in calculations). All FMC values were reviewed to ensure that the formatting was consistent. Data were plotted to check for outliers, and two outlying FMC values were identified and removed which fell widely outside the range of FMC for all other samples in a given fuel (>600% FMC in a live heather sample, >400% in a dead gorse sample). The moisture content of eleven samples was below 0. The FMC of seven of these was between −2% and 0% and the FMC of four was lower than −20%. We therefore selected −2% FMC as the threshold below which data were discarded, as negative values above this threshold were likely due to scale error. These six values are marked as outliers in the UK-wide dataset. No outliers were removed from the NYM or WM dataset as these data appeared normally distributed. Samples were collected by several individuals, so data were compiled and sample site names were standardised.

## Supplementary information

Supplementary file

## Data Availability

No custom computer code or algorithms were used to process or generate the data presented in this manuscript.
